# Follower Mindfulness and Well-Being: The Mediating Role of Perceived Authentic Leadership and the Moderating Role of Leader Mindfulness

**DOI:** 10.3389/fpsyg.2020.00879

**Published:** 2020-05-20

**Authors:** Jing Zhang, Lynda J. Song, Dan Ni, Xiaoming Zheng

**Affiliations:** ^1^Department of Human Resources Management, Hebei University of Economics and Business, Shijiazhuang, China; ^2^Management Division, University of Leeds, Leeds, United Kingdom; ^3^Department of Leadership and Organization Management, Tsinghua University, Beijing, China

**Keywords:** leader mindfulness, follower mindfulness, perceived authentic leadership, well-being, implicit leadership theory

## Abstract

Drawing on implicit leadership theory and the mindfulness literature, we propose that perceived authentic leadership mediates the relationship between follower mindfulness and follower well-being. Leader mindfulness plays a moderating role in this process. We validated these hypotheses with the two-wave data from 56 leaders and 275 followers in two private enterprises located in China. We used Mplus 8.0 to test our hypotheses. Consistent with our hypotheses, the results showed that perceived authentic leadership mediated the positive relationship between follower mindfulness and follower well-being. Higher leader mindfulness enhanced the effect of follower mindfulness on perceived authentic leadership and also strengthened the indirect effect of follower mindfulness on follower well-being via perceived authentic leadership. The theoretical and managerial implications are further discussed in the light of these findings.

## Introduction

In the Internet era, many competition and challenges seem to be inevitable and pervasive within organizational life, which has brought immense pressure to bear on members of those organizations. For instance, the huge amounts of information, faster work speed, and countless interruptions faced by today’s workers may all directly damage employee well-being. In turn, the issue of how to improve employee well-being has attracted growing attention from both scholars and managers.

As a crucial approach to improving individual well-being, mindfulness practices have been integrated into many therapies in the fields of psychology and clinical medicine (Brown and Ryan, 2003; Carmody and Baer, 2008). A large body of work suggests that mindfulness effectively relieves stress and enhances well-being (Hülsheger et al., 2013; Schultz et al., 2015). These existing research findings have greatly promoted the application of mindfulness in the workplace. For instance, Google, General Mills, and Procter and Gamble have offered “mindfulness” courses (Tan, 2012; Wolever et al., 2012) to improve employees’ working states and seek sustainable development as a whole.

Mindfulness, defined in general terms as “paying attention in a particular way: on purpose, in the present moment, and non-judgmentally” (Kabat-Zinn, 2005, p. 4), is characterized by full attention to the present, high awareness to the internal and the external, and acceptance with non-judgment (Brown and Ryan, 2003; Baer et al., 2004). Recently, Glomb et al. (2011) regard mindfulness as receptive attention to and awareness of present events and experience, including both internal (e.g., thoughts and sensations) and external (e.g., social environment) stimuli. Although research has verified the positive relationship between individual mindfulness and well-being (Good et al., 2016), it is little known about the mediating mechanisms in this process. More importantly, we are not clear about the interactive effect of follower and leader mindfulness on follower well-being. This is a critical issue because followers interact closely with their leaders during each workday (Uhl-Bien et al., 2014). Hence, leaders’ characteristics are expected to be important situational factors that affect followers’ psychological states (Avolio et al., 2009). If we look at only one side, it is hard to obtain a comprehensive understanding of the effect of mindfulness on well-being because of the importance of both follower and leader characteristics in influencing leadership perception (Felfe and Schyns, 2010). For example, exclusive focus on the follower side overemphasizes how the picture in mind of followers plays a role in leadership perception, leading to inaccurate knowledge and even misunderstanding in this regard.

**FIGURE 1:**
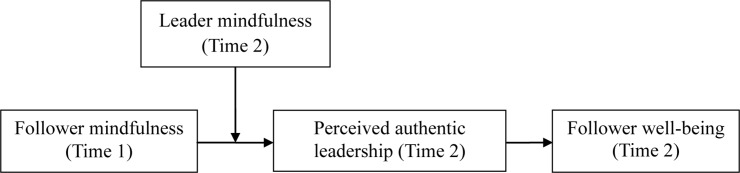
The hypothesized model.

Based on implicit leadership theory and the mindfulness literature, this study aims to examine *how* and *when* follower mindfulness influences well-being. Follower personality becomes an important factor influencing how followers perceive leader behaviors, and it further exerts an impact on followers’ outcomes (Nye, 2002). In this study, we focus on perceived authentic leadership—a pattern of leader behavior that draws upon and promotes both positive psychological capacities and a positive ethical climate, to foster greater self-awareness, an internalized moral perspective, balanced processing of information, and relational transparency on the part of leaders working with followers, fostering positive self-development” (Walumbwa et al., 2008, p. 94) – as an important mediator. The feature of follower mindfulness is considered foundational for perceived authentic leadership (Kinsler, 2014) because it helps followers construct the leadership prototype of authentic leadership and in turn improves their well-being. More importantly, in addition to follower characteristics, leaders are particularly important constituencies in the emergence process of leadership perception and affect how followers perceive leader behaviors (Lord et al., 1984; Van Quaquebeke et al., 2011). In general, followers regard leaders who are similar to themselves as ideal leaders (Keller, 1999). Accordingly, we expect that higher leader mindfulness improves the effect follower mindfulness on perceived authentic leadership. Taken together, our study proposes a moderated mediation model that leader mindfulness moderates the indirect effect of follower mindfulness on follower well-being through perceived authentic leadership (see Figure 1).

By doing so, our study extends the mindfulness literature by providing a new explanation for the influence of mindfulness on well-being. First, we elaborate on *how* follower mindfulness influences well-being by considering perceived authentic leadership as a mediator. Prior studies have not yet provided adequate theoretical and empirical evidence on this regard, whereas the investigation of a new underlying mechanism sheds additional light on the link between mindfulness and well-being, thereby enriching the mindfulness literature. Meanwhile, we suggest that one’s personality (i.e., mindfulness) plays a role in forming the perception of authentic leadership, which highlights the importance of mindfulness in constructing an ideal leadership prototype and influencing their access to leader behaviors. In doing so, we respond to Reb et al.’s (2014) call for scholars to “examine the relation between mindfulness and authentic leadership” (p. 43) in the workplace. Second, to our knowledge, little research has sought to combine follower and leader mindfulness to examine their influences on follower outcomes. In consequence, we are unclear about whether a substitution effect or a joint effect occurs between follower mindfulness and leader mindfulness. The new perspective—combining two parties’ mindfulness—is valuable because it helps us gain a deeper understanding of *when* follower and leader mindfulness would exert a more powerful effect to improve follower well-being. Third, our study builds bridges between the research on leadership and the mindfulness literature by integrating implicit leadership theory as the overarching theoretical framework and applying it into the research on mindfulness and authentic leadership. We suggest that both leader and follower dispositional mindfulness are important constituencies of the leadership process, which facilitates a better understanding of authentic leadership perception as the outcome of the interactive effects of leader and follower mindfulness.

## Theory and Hypotheses

### Implicit Leadership Theory

Implicit leadership theory suggests that followers use a cognitive categorization system during information processing to encode specific leader behaviors, and an implicit leadership prototype acts as an intrinsic reference for judging the leader (Shondrick et al., 2010). The leadership prototype and perceptions depend largely on followers’ personality and interpretations (Offermann et al., 1994; Kenney et al., 1996; Nye, 2002). Therefore, the importance of followers’ perceptions of leadership emphasized in implicit leadership theory provides a theoretical basis for exploring how followers’ personalities affect their perceptions of specific leader behavior. Additionally, the degree to which the leader’s personality matches an ideal stereotype constructed in the followers’ imagination (Van Knippenberg et al., 2006; Giessner et al., 2009) influences followers’ perceptions of certain leadership behaviors and, in turn, their psychological and behavioral outcomes at work.

### The Mediating Role of Perceived Authentic Leadership

Perceived authentic leadership reflects that followers experience greater self-awareness, relational transparency, an internalized moral perspective, and balanced processing of information from their leaders (Walumbwa et al., 2008). On the basis of implicit leadership theory and the characteristics of mindfulness, we suggest that follower mindfulness enhances followers’ perceptions of authentic leadership.

Mindful followers are characterized by full attention to the present, high awareness to the internal and the external, and acceptance with non-judgment (Baer et al., 2004). Research has shown that employees with high mindfulness would exhibit more authentic behaviors (Leroy et al., 2013). Mindful followers are also sensitive to authentic behaviors of leaders, expecting leaders to be fully aware of the environment and treat them honestly and openly. As such, it is plausible for them to recognize authentic leadership as an ideal leadership prototype, which influences how followers access to and process leader behaviors. For example, mindful followers with better awareness, attention, and acceptance tend to have a clearer and closer observation of leaders’ words and deeds and to accept the true thoughts and feelings expressed by leaders. Hence, mindful followers are expected to have a more acute perception of the characteristics related to authentic leadership.

The above theorizing and evidence suggest that mindful followers may regard authentic leadership as the prototype of implicit leadership and thus have a higher level of perceived authentic leadership. Therefore, we hypothesize:

Hypothesis 1: Follower mindfulness is positively related to perceived authentic leadership.

Implicit leadership theory indicates that followers’ perceptions of leader behaviors exert an effect on follower outcomes (Van Quaquebeke et al., 2011). In this study, we focus on followers’ well-being—defined as mental well-being, including positive affect and psychological functioning (autonomy, competence, self-acceptance, and personal growth) and interpersonal relationships (Tennant et al., 2007).

When followers perceive a high level of authentic leadership, they would not only be deeply aware of leaders’ consistency and trustworthiness in their words and actions but also experience adequate support in growth and development from leaders (Wang and Hsieh, 2013; Kiersch and Byrne, 2015). Relatedly, this internalized moral perspective embedded in perceptions of authentic leadership enhances followers’ psychological functioning (Al Zaabi et al., 2016). Meanwhile, high perceptions of authentic leadership reflect that followers experience unbiased processing and self-awareness of leaders. It improves the self-acceptance and personal growth of those followers (Wang et al., 2014), both of which are related to individual well-being. The relational transparency and authentic relational orientation of perceived authentic leadership may also serve as precursors to positive interpersonal relationships and followers’ positive emotions (Ilies et al., 2005). Overall, such perceived authenticity can create a positive context for enhancing followers’ well-being (Rahimnia and Sharifirad, 2015).

Empirically, research indicated that perceived authentic leadership improves follower well-being. For example, Rahimnia and Sharifirad (2015) found that perceived authentic leadership increased job satisfaction and reduced stress as indicators of well-being. Gardner et al. (2005) indicated that positive modeling of authentic leaders increased followers’ internalized regulation processes, leading to higher well-being. The preceding argument and evidence suggest that followers who perceive high levels of authentic leadership are expected to experience enhanced well-being. Taken together, combining with Hypothesis 1, we hypothesize:

Hypothesis 2: Perceived authentic leadership mediates the relationship between follower mindfulness and follower well-being.

### The Moderating Role of Leader Mindfulness

Drawing on implicit leadership theory (Keller, 1999), we further propose that leader mindfulness is a boundary condition in the relationship between follower mindfulness and perceived authentic leadership. Implicit leadership theory suggests that the process of follower forming the perceptions of certain leadership largely depends on leader characteristics. When leaders match an ideal stereotype resulting from followers’ imaginary one (Van Knippenberg et al., 2006; Giessner et al., 2009), followers are more likely to have an improved perception of this leadership. Thus, we suggest that leaders with high mindfulness are more likely to be in accord with mindful followers’ leader stereotype, which thus strengthens the effect of follower mindfulness and perceived authentic leadership.

Specifically, leaders with high mindfulness have clearer self-awareness and higher flexibility in awareness, reducing their propensity toward automatic responses (Bishop et al., 2004). It can also help leaders obtain more accurate information and responses from their followers (Lakey et al., 2008). Relatedly, by keeping clearer self-awareness and higher flexibility in awareness (Baer et al., 2004), as well as keeping their full attention directed to the current situation and engaging in continual communication with their followers, mindful leaders are more likely to not only have more effective insights and responses to the followers’ requirements during their interactions but also obtain more accurate information and responses (Lakey et al., 2008). Under this condition, those high-mindful followers are more likely to capture mindful leaders’ desirable characteristics such as self-awareness and self-concordant moral perspective that are key to form authentic leadership, thus perceiving a high level of authentic leadership. Conversely, those low-mindful followers, who can hardly keep attention and awareness to the mindful characteristics of their leaders without judgment, may not have sufficient perceptions of authentic leadership.

Moreover, leaders with high mindfulness are open and accepting of others, so they tend to treat others honestly and openly and to express their true selves and share their truthful feelings with their followers (Kernis and Goldman, 2006). As leaders with high mindfulness foster a receptive attitude toward information, their high-mindful followers are more likely to perceive the leader as demonstrating the unbiased processing of information (Emanuel et al., 2010) and truthful communications that represent the core contents of authentic leadership. In addition, given that high-mindful followers have stable attention and non-judgmental acceptance attitudes (Beckman et al., 2012), they tend to clearly witness, capture, and accept the true thoughts and feelings expressed by leaders with non-judgment, leading to a high level of perceived authentic behaviors. By contrast, low-mindful followers can hardly keep an open and receptive attitude and high attention to these characteristics of their leaders and accordingly may perceive a low level of authentic leadership.

In contrast, leaders with low mindfulness are not fully aware of and attentive to the present moment without judgment. Such leaders find it difficult to not only give accurate feedback and pay adequate attention when communicating with followers but also express their true selves and feelings with others. Consequently, they cannot effectively understand and capture their followers’ needs, which leads to both communication problems and social distance (Green et al., 1996). Meanwhile, their tendency of automatic processing prevents them from considering options that might be more closely aligned to their needs and values (Ryan et al., 1997). Such characteristics of low mindfulness run contrary to authentic leadership as an ideal stereotype that their followers imagine. As such, the positive effect of follower mindfulness on perception of authentic leadership is diminished.

In summary, these arguments and empirical evidence suggest that leader mindfulness moderates the relationship between follower mindfulness and perceived authentic leadership. Therefore, we hypothesize:

Hypothesis 3: Leader mindfulness moderates the relationship between follower mindfulness and perceived authentic leadership such that the positive relationship between follower mindfulness and perceived authentic leadership is strengthened when leader mindfulness is higher rather than lower.

### The Moderated Mediation Model

Combining Hypotheses 1, 2, and 3, we therefore expect an integrative model that leader mindfulness moderates the meditating effect of perceived authentic leadership on the relationship between follower mindfulness and follower well-being. That is, faced with highly mindful leaders, followers with a high level of mindfulness would be more likely to perceive more authentic leadership and, therefore, to experience enhanced well-being. In contrast, interacting with a leader who has a low level of mindfulness, mindful followers would be less likely to experience authentic leadership and, in turn, tend to have a low level of well-being. On the basis of on the above arguments, we propose:

Hypothesis 4: Leader mindfulness moderates the indirect effect of follower mindfulness on follower well-being via perceived authentic leadership such that the indirect effect is strengthened when leader mindfulness is higher rather than lower.

## Materials and Methods

### Sample and Procedures

Our sample consisted of full-time workers who were working in two manufacturing companies – a household appliances listed company and a petrochemical equipment company – located in China. With the assistance of their human resources departments, we invited 66 team leaders and 320 followers to take part in the study. We collected our data from two sources (i.e., leaders and followers) at two time points across 2 months so as to minimize common method variance (Podsakoff et al., 2012). At Time 1, followers were asked to report their dispositional mindfulness and demographic information. Leaders also reported their demographics. We received 65 leader questionnaires (98.48% response rate) and 314 follower questionnaires (98.12% response rate). Two months later (Time 2), we asked followers to report their well-being and perceived authentic leadership. Leaders were asked to report their dispositional mindfulness. We received 65 leadership questionnaires (98.48% response rate) and 284 follower questionnaires (88.75% response rate) during this phase of the study. Nine teams that had only one follower were excluded. Finally, we obtained the data from the 56 team leaders and 275 followers. In the final sample, 64.3% of the leaders were men; their average age was 39.95 years (*SD* = 6.70). Meanwhile, 55.6% of the followers were men; their average age was 35.59 years (*SD* = 7.64). The followers average tenure in the organization was 6.04 years (*SD* = 86.55).

### Measures

We adopted a standard translation/back-translation procedure to translate previously published English scales into the Chinese versions (Brislin, 1980). For dispositional mindfulness, we used a six-point Likert scale ranging from 1 (“almost never”) to 6 (“almost always”). For other measures, we used six-point Likert scales ranging from 1 (“strongly disagree”) to 6 (“strongly agree”).

#### Dispositional Mindfulness

Following the previous studies, we consider mindfulness as a dispositional construct that is more highly stable in reflecting individual differences and predicting outcomes (Brown et al., 2007; Hülsheger et al., 2013). Both follower and leader dispositional mindfulness was assessed using Brown and Ryan’s (2003) 15-item Mindful Attention Awareness Scale. Participants were asked to indicate how frequently or infrequently they had the everyday experiences described in each statement. Sample items are “I find it difficult to stay focused on what’s happening in the present” (reverse coded), “I do jobs or tasks automatically, without being aware of what I’m doing” (reverse coded), and “I find myself preoccupied with the future or the past” (reverse coded). Cronbach’s alpha for the leader mindfulness scale was 0.88; that for the follower mindfulness scale was 0.76.

#### Perceived Authentic Leadership

Perceived authentic leadership was assessed using Neider and Schriesheim’s (2011) 16-item Authentic Leadership Inventory. This scale contained four subdimensions of authentic leadership: self-awareness (α = 0.78; a sample item of four items is “She/he clearly knows his/her likes and dislikes”), relational transparency (α = 0.83; a sample item of four items is “She/he shares his/her feelings with others”), internalized moral perspective (α = 0.84; a sample item of four item is “She/he is guided in his/her actions by internal moral standards”), and balanced processing (α = 0.89; a sample item of four items is “She/he asks for ideas that challenge his/her core beliefs”). Cronbach’s alpha for the 16-item scale was 0.95.

#### Well-Being

Well-being was measured using the 14-item scale developed by Tennant et al. (2007). Sample items are “I have been feeling optimistic about the future” and “I have been feeling useful.” Cronbach’s alpha for this scale was 0.92.

#### Control Variables

We included follower demographics (i.e., age, gender, and tenure in organization) and leader demographics (i.e., age and gender) as control variables. First, there is the theoretical and empirical basis for assuming that those variables have particular connections to the focal variables. Theoretically, we controlled follower age because age influences individual capacity to self-express (Harter, 2002), which affects interaction quality between followers and leaders and ultimately the followers’ perceptions of authentic leadership. We included age as a control variable because of the significant relationship between age and well-being (Allen et al., 2017). Follower demographics such as gender were considered, as previous studies suggest that gender exerts large effects on followers’ prototypes of authentic leadership (Monzani et al., 2015). Empirically, individual age, gender, and tenure in organization were widely controlled in most studies of authentic leadership and well-being (e.g., Reb et al., 2013; Sendjaya et al., 2016; Pinck and Sonnentag, 2018). For leader demographics, given that little research on leader mindfulness and follower well-being, we followed Reb et al.’s (2014) study to control leader age and gender. According to Spector and Brannick’s (2011) and Bernerth and Aguinis’s (2016) recommendations, we included such control variables to exclude the alternative hypotheses that leader and follower demographics rather than mindfulness play a role in predicting perceived authentic leadership.

### Analytical Strategy

We first obtained the means, standard deviances, and intercorrelations for our study variables at both the within-team and between-team levels. Next, we performed multilevel confirmatory factor analyses to examine the discriminative validity of the study variables. Given the nested structure of our data, we used multilevel structural equation modeling in Mplus 8.0 (Muthén and Muthén, (1998–2018)). Before the analysis, we group-mean centered follower age, gender, tenure in organization, and the independent variable (i.e., follower mindfulness); and we grand-mean centered leader gender, age, and leader mindfulness; the intention was to separate the cross-level interaction from the between-team interaction to avoid detecting a spurious cross-level effect (Hofmann and Gavin, 1998). The Monte Carlo method recommended by Preacher et al. (2010) was used to estimate confidence intervals (CIs) for the hypothesized mediated relationship to determine their significance. The cross-level interaction effect was tested in path-analytical models with a moderator at the team level. To test the mediated moderation hypothesis, we used Monte Carlo simulation to test the cross-level conditional indirect effect (Bauer et al., 2006).

## Results

### Descriptive Statistics

Table 1 shows the means, standard deviations, and correlations for the variables at the within-team and between-team levels in the study and the alpha coefficients. At the within-team level, follower mindfulness was positively related to perceived authentic leadership (*r* = 0.20, *p* < 0.001) and follower well-being (*r* = 0.21, *p* < 0.001). Perceived authentic leadership was positively related to follower well-being (*r* = 0.40, *p* < 0.001). The results provided initial evidence in support of our hypothesized relationships.

**TABLE 1:** Means, standard deviations, and correlations among variables.

Variables	Mean	SD_within–team_	SD_between–team_	1	2	3	4	5	6	7	8	9
1. Follower age	35.50	6.91	3.20	–	–0.19	0.87***	0.40**	0.19	–0.05	0.67***	0.15	0.27*
2. Follower gender	0.46	0.41	0.28	0.09	–	−0.43**	−0.55***	−0.52***	−0.77***	–0.19	0.40**	-0.04
3. Follower tenure in organization	72.75	49.99	31.18	0.32***	0.19**	–	0.51***	0.62***	0.36**	0.73***	0.01	0.26
4. Follower mindfulness (T1)	4.63	0.56	0.13	–0.01	0.04	0.01	(0.76)	0.31*	0.55***	0.67***	–0.06	0.53***
5. Perceived authentic leadership (T2)	4.47	0.73	0.30	–0.10	0.06	−0.19**	0.20***	(0.95)	0.81***	0.41**	–0.14	0.02
6. Follower well−being (T2)	4.45	0.61	0.14	–0.01	0.01	−0.14*	0.21***	0.40***	(0.92)	0.18	−0.27*	0.23
7. Leader age	39.95	6.64	6.64							–	0.17	0.07
8. Leader gender	0.36	0.48	0.48								–	0.08
9. Leader mindfulness (T2)	4.70	0.66	0.63									(0.88)

*n* = 275 at the individual level and *N* = 56 at the team level. Correlations above the diagonal represent between-team (aggregated) scores. Correlations below the diagonal represent within-team scores. Cronbach’s alphas are reported in the parentheses on the diagonal. ***p < 0.001; **p < 0.01; *p < 0.05 (two-tailed).

### Confirmatory Factor Analysis

We conducted a multilevel confirmatory factor analysis to test the discriminative validity of the focal variables in our model. To examine the discriminant validity, we compared the hypothesized four-factor model with four alternative models. Given that the ratio of the sample size and the total number of items impairs overall model fit, scholars suggest that using item parcels should reduce the number of parameters and mitigate the impairment (Little et al., 2013). We parceled the constructs of follower mindfulness, follower well-being, and leader mindfulness into three items using the item-to-construct balance approach (Little et al., 2002). Given that authentic leadership is a multidimensional construct, we parceled it using the domain-representative approach that parcels were created by joining items from each dimension into item sets (Kishton and Widaman, 1994). The results showed that the hypothesized four-factor model (i.e., follower mindfulness, perceived authentic leadership, follower well-being, and leader mindfulness) provided a good fit to the data [χ^2^ = 143.52; *df* = 91; SRMR_within–team_ = 0.02; SRMR_between–team_ = 0.19; root mean square error of approximation (RMSEA) = 0.05, comparative fit index (CFI) = 0.98; Tucker–Lewis index (TLI) = 0.97]. It produced significant improvements in model fit over alternative models (see Table 2). These results provide support for the discriminative validity of the four constructs.

**TABLE 2:** Results of confirmatory factor analysis.

Models	χ^2^	*df*	Δχ^2^	χ^2^/*df*	SRMR_within–team_	SRMR_between–team_	RMSEA	CFI	TLI
A four-factor model (fm; fal; fwb; lm)	143.52	91		1.58	0.02	0.19	0.05	0.98	0.97
A first three-factor model (fm and fal; fwb; lm)	450.49	96	306.97***	4.69	0.12	0.58	0.12	0.87	0.83
A second three-factor model (fm and fwb; fal; lm)	481.10	96	337.58***	5.01	0.12	0.30	0.12	0.85	0.81
A third three-factor model (fal and fwb; fm; lm)	1,128.70	96	985.18***	11.76	0.16	0.41	0.20	0.61	0.50
A two-factor model (fm, fal, and fwb; lm)	776.48	99	632.96***	7.84	0.19	0.44	0.16	0.74	0.68

*n* = 275 at the individual level; *N* = 56 at the team level. Δχ^2^ values were compared with the hypothesized four-factor model. fm, follower mindfulness; fal, perceived authentic leadership; fwb, follower well-being; lm, leader mindfulness; RMSEA, root mean square error of approximation; CFI, comparative fit index; TLI, Tucker–Lewis index. ***p < 0.001 (two-tailed).

### Hypothesis Testing

Table 3 presents the results of our hypothesis testing. Hypothesis 1 proposes that follower mindfulness is positively related to perceived authentic leadership. Consistent with this hypothesis, the results showed that the relationship between follower mindfulness and perceived authentic leadership was significant (γ = 0.30, *p* < 0.01). Hypothesis 2 proposed that the indirect effect of follower mindfulness on follower well-being is mediated by perceived authentic leadership. The results showed that the 95% CI for the indirect effect did not include zero [γ = 0.09, 95% CI = (0.02, 0.19)], supporting Hypothesis 2.

**TABLE 3:** Results of multilevel path analyses.

	Perceived authentic leadership	Follower well-being
Follower age	−0.00 (0.01)	
Follower gender	0.15 (0.01)	
Follower tenure in organization	−0.00*** (0.00)	
Follower mindfulness	0.30** (0.11)	
Perceived authentic leadership		0.32*** (0.07)
Leader age	0.02* (0.01)	−0.00 (0.01)
Leader gender	−0.14 (0.12)	−0.03 (0.06)
Leader mindfulness	0.01 (0.08)	
Follower mindfulness × Leader mindfulness	0.22** (0.06)	

Standard errors (SEs) of the coefficients are shown in the brackets. Follower age, gender, tenure in organization, and mindfulness were person-mean centered; leader age, gender, and mindfulness were grand-mean centered. Without any control variables in the model, our hypotheses were still supported. ***p < 0.001; **p < 0.01; *p < 0.05 (two-tailed).

**FIGURE 2:**
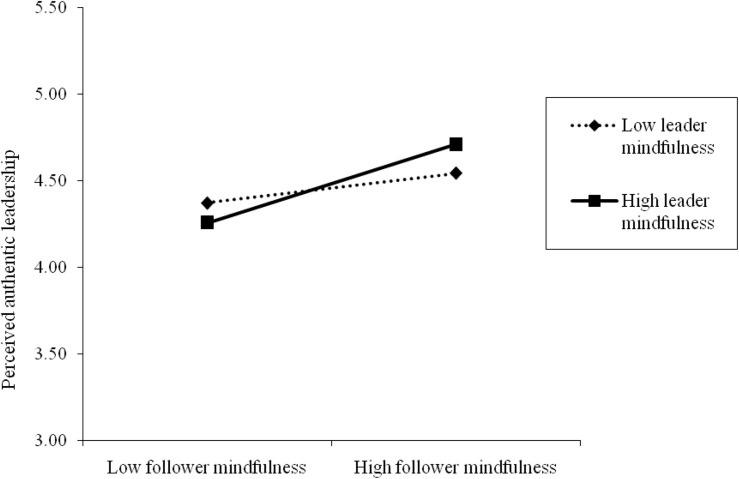
The interaction effect of follower mindfulness and leader mindfulness on perceived authentic leadership.

Hypothesis 3 predicted that leader mindfulness moderates the relationship between follower mindfulness and perceived authentic leadership. We found that the interaction effect of follower mindfulness and leader mindfulness on perceived authentic leadership was significant (γ = 0.22, *p* < 0.01). We plotted this interaction effect at conditional values of leader mindfulness (see Figure 2). When we conducted a simple slope analysis, as recommended by Preacher et al. (2006), the results showed that the positive relationship between follower mindfulness and perceived authentic leadership was significant at higher levels (i.e., +1 *SD*) of leader mindfulness (simple slope = 0.43, *t* = 3.78, *p* < 0.001). Conversely, at lower levels (i.e., –1 *SD*) of leader mindfulness, this positive relationship was not significant (simple slope = 0.16, *t* = 1.42, *n.s.*). Therefore, Hypothesis 3 was supported.

Hypothesis 4 proposed that leader mindfulness moderates the mediation effect of follower mindfulness on follower well-being via perceived authentic leadership. We tested the conditional indirect effect at two levels of leader mindfulness (+1 *SD* and −1 *SD*). The results indicated that the indirect effect was significant with a high level of leader mindfulness [γ = 0.14, 95% CI = (0.05, 0.26)] but was not significant with a low level of leader mindfulness [γ = 0.05, 95% CI = (−0.01, 0.13)]. Furthermore, the difference in these indirect effects was significant [γ = 0.09, 95% CI = (0.03, 0.16)], supporting Hypothesis 4.

We estimated values of pseudo *R*-square to assess the amount of incremental variance in the mediator and outcome variables explained by the study variables. We found that the moderated mediation model explained 9.11% of the variance in perceived authentic leadership and 17.07% of the variance in follower well-being.

## Discussion

The moderated mediation model was supported. We found that follower mindfulness was positively related to perceived authentic leadership and in turn positively related to follower well-being. High leader mindfulness enhanced the effect of follower mindfulness on perceived authentic leadership and the indirect effect of follower mindfulness on their well-being via perceived authentic leadership. Accordingly, this study not only contributes to the mindfulness and leadership literatures but also provides significant management guidelines for practice.

### Theoretical Contributions

First, we unveil an important and distinct mechanism to explain the effect of follower mindfulness on well-being by integrating perceived authentic leadership as a mediator. Our research showed that follower perceived authentic leadership transmitted the indirect of follower mindfulness on follower well-being. To our best knowledge, previous studies exclusively focused on such perspectives as psychological need satisfaction, positive job-related affect, and psychological capital to examine how individual mindfulness influences well-being (Malinowski and Lim, 2015), but they ignored the leadership perception as a mediator. We believe that this investigation on the mechanisms of the mindfulness effects from the perspective of the perceived leadership responds to scholars’ suggestions that the future research should deepen the study of mindfulness and leadership in the workplace (Lippincott, 2018), especially in regard to the link between mindfulness and authentic leadership (Reb et al., 2014). Meanwhile, our examination in this study also enriches the existing dialogue regarding mindfulness and leadership (e.g., Walsh and Arnold, 2018; Arendt et al., 2019; Schuh et al., 2019).

Second, we contribute to the mindfulness literature in the workplace by examining the interactive effect of follower and leader mindfulness on follower well-being via perceived authentic leadership. Previous studies have highlighted the importance of mindfulness for individual well-being, but most did not consider the effects of both actors’ mindfulness, which inhibits our comprehensive understanding of the effectiveness of mindfulness. For example, such investigation leaves unsettled the question of whether there is a substitution effect or a joint effect between follower mindfulness and leader mindfulness. By examining leader mindfulness as a moderator, our study discovered that the positive relationship between follower mindfulness and perceived authentic leadership was strengthened when leader mindfulness was higher rather than lower, which further improved followers’ well-being. Such shift from a traditional approach to an interactive approach is necessary to fully account for the role of mindfulness within organizations. As a result, these findings help us gain a deep understanding of when follower mindfulness in organizations can predict better outcomes.

Third, we extend the literature on authentic leadership by showing that perceived authentic leadership was influenced by both leader and follower mindfulness and, in turn, has an impact on follower well-being. Our study verified that taking both follower mindfulness and leader mindfulness into consideration allows us to more accurately examine how authentic leadership forms in the workplace. Meanwhile, we offered empirical evidence of the positive effect of authentic leadership on well-being, thereby promoting the dialogue about the effectiveness of authentic leadership on followers’ outcomes.

Finally, our findings advance implicit leadership theory by applying it to the mindfulness literature as well as the authentic leadership literature. We clearly explain how leadership perception (i.e., perceived authentic leadership) is formed from both follower-centered and leader-centered perspectives. By doing so, this study develops the application domain of this theory and offers a more comprehensive understanding of mindfulness by exploring research areas that have not been tapped fully by implicit leadership theory.

### Practical Implications

The first implication relates to its revelation that organizations should encourage employees to be aware of the importance of being mindful at work, because a high level of mindfulness was positively associated with the beneficial outcome (i.e., well-being). Research has shown that individual mindfulness can be improved by mindfulness training over a given period of time (Leroy et al., 2013). Even a short-term mindfulness intervention has a significant impact in terms of behavioral changes. Thus, organizations could encourage employees to do mindfulness exercises in the workplace to maintain well-being through spontaneous or prearranged ways (Hülsheger et al., 2015). Also, organizations can introduce mindfulness training programs such as mindfulness-based stress reduction and mindfulness-based cognitive therapy that are widely recognized and accepted in practice (Kabat-Zinn, 2005) to promote individual mindfulness.

More importantly, both followers and leaders with a high level of mindfulness can potentially promote follower well-being through perceived authentic leadership. Therefore, mindful features should be taken into consideration in the process of personnel selection. In terms of team composition, organizations should consider the match between follower mindfulness and leader mindfulness as a means to increase employee well-being. For example, for especially mindful followers (as compared with non-mindful followers), it is appropriate for organizations to match them with a mindful leader so as to bring about better outcomes. In light of the importance of their mindfulness for follower well-being, both employees and leaders should actively strive to cultivate their personal mindfulness through formal and informal mindfulness exercises.

Additionally, given the positive effect of perceived authentic leadership on well-being in this study, we suggest that organizations should improve leaders’ authentic leadership behaviors by providing feasible and useful leadership training. For instance, organizations can offer mindfulness training to develop authentic leadership (Leroy et al., 2015).

### Limitations and Future Directions

Despite these strengths, our study has several limitations that suggest possible avenues for future research. Its first limitation is that the data were collected from two companies in China. Given the differences among different enterprises, industries, and areas, the external validity of the conclusions might be impaired by the samples selected. Although our findings offer important and unique insights on mindfulness in the Chinese context and have significant implications for Chinese practice, we suggest future research should test our hypotheses using larger samples in different enterprises, industries, and countries.

Second, we tested perceived authentic leadership as one important mediator of the interactive effect of follower and leader mindfulness on follower well-being, but alternative mechanisms might potentially exist. For instance, previous studies have found overlaps between authentic leadership and other leadership behaviors, such as ethical leadership, and servant leadership (Hoch et al., 2018). Therefore, to discover the unique effect of mindfulness on well-being beyond the existing explanations, we suggest future research investigate other potential mechanisms underlying the relationship between mindfulness and well-being.

Finally, the non-experimental nature of our study prevented us from making strong causal inferences, although we conducted a multiple-wave survey that may remove some ambiguities about the direction of causation. Future research might consider conducting experimental studies in both the field and the laboratory or adopt a longitudinal research design to better capture the causality among those variables.

## Conclusion

This study links the mindfulness literature to the leadership research by examining the mediating role of perceived authentic leadership and the moderating role of leader mindfulness in the relationship between follower mindfulness and follower well-being. The current study provides a new explanation for how and when mindfulness influences individual well-being in the workplace, and it offers important implications for practitioners.

## Data Availability Statement

The datasets generated for this study are available on request to the corresponding author.

## Ethics Statement

This research was carried out in accordance with the ethical guidelines of the American Psychological Association. On the cover pages of questionnaires and the introduction of the research, all participants were informed of the research purposes, their freedom of participating in and quitting the research, and the assurance of the confidentiality.

## Author Contributions

All authors listed have made a substantial, direct and intellectual contribution to the work, and approved it for publication.

## Conflict of Interest

The authors declare that the research was conducted in the absence of any commercial or financial relationships that could be construed as a potential conflict of interest.
